# Untargeted metabolomic, and proteomic analysis identifies metabolic biomarkers and pathway alterations in individuals with 22q11.2 deletion syndrome

**DOI:** 10.1007/s11306-024-02088-0

**Published:** 2024-02-28

**Authors:** Marwa Zafarullah, Kathleen Angkustsiri, Austin Quach, Seungjun Yeo, Blythe P. Durbin-Johnson, Heather Bowling, Flora Tassone

**Affiliations:** 1 Department of Biochemistry and Molecular Medicine, School of Medicine, University of California Davis, Sacramento, CA 95817 USA; 2 Department of Pediatrics, School of Medicine, University of California Davis, Sacramento, CA 95817 USA; 3 Dalton Bioanalytics Inc, Los Angeles, CA USA; 4 Division of Biostatistics, School of Medicine, University of California, Davis, CA USA; 5 Epistemic AI, Westport, CT USA; 6 MIND Institute, University of California Davis Medical Center, Sacramento, CA 95817 USA

**Keywords:** 22q11.2 deletion syndrome, Proteomics, Metabolomics, Biomarker, Pathways, APS, AS

## Abstract

**Introduction:**

The chromosome 22q11.2 deletion syndrome (22q11.2DS) is characterized by a well-defined microdeletion and is associated with a wide range of brain-related phenotypes including schizophrenia spectrum disorders (SCZ), autism spectrum disorders (ASD), anxiety disorders and attention deficit disorders (ADHD). The typically deleted region in 22q11.2DS contains multiple genes which haploinsufficiency has the potential of altering the protein and the metabolic profiles.

**Objectives:**

Alteration in metabolic processes and downstream protein pathways during the early brain development may help to explain the increased prevalence of the observed neurodevelopmental phenotypes in 22q11.2DS. However, relatively little is known about the correlation of dysregulated protein/metabolite expression and neurobehavioral impairments in individuals who developed them over time.

**Methods:**

In this study, we performed untargeted metabolic and proteomic analysis in plasma samples derived from 30 subjects including 16 participants with 22q11.2DS and 14 healthy controls (TD) enrolled in a longitudinal study, aiming to identify a metabolic and protein signature informing about the underlying mechanisms involved in disease development and progression. The metabolic and proteomic profiles were also compared between the participants with 22q11.2DS with and without various comorbidities, such as medical involvement, psychiatric conditions, and autism spectrum disorder (ASD) to detect potential changes among multiple specimens, collected overtime, with the aim to understand the basic underlying mechanisms involved in disease development and progression.

**Results:**

We observed a large number of statistically significant differences in metabolites between the two groups. Among them, the levels of taurine and arachidonic acid were significantly lower in 22q11.2DS compared to the TD group. In addition, we identified 16 proteins that showed significant changes in expression levels (adjusted P < 0.05) in 22q11.2DS as compared to TD, including those involved in 70 pathways such as gene expression, the PI3K-Akt signaling pathway and the complement system. Within participants with 22q11.2DS, no significant changes in those with and without medical or psychiatric conditions were observed.

**Conclusion:**

To our knowledge, this is the first report on plasma metabolic and proteomic profiling and on the identification of unique biomarkers in 22q11.2DS. These findings may suggest the potential role of the identified metabolites and proteins as biomarkers for the onset of comorbid conditions in 22q11.2DS. Ultimately, the altered protein pathways in 22q11.2DS may provide insights of the biological mechanisms underlying the neurodevelopmental phenotype and may provide missing molecular outcome measures in future clinical trials to assess early-diagnosis treatment and the efficacy of response to targeted treatment.

**Supplementary Information:**

The online version contains supplementary material available at 10.1007/s11306-024-02088-0.

## Introduction

A hemizygous deletion of approximately 40 contiguous genes on chromosome 22 causes chromosome 22qDS11.2 deletion syndrome (22qDS) also called Velo-cardio-facial syndrome (VCFS) (Shprintzen et al., 2005) or DiGeorge syndrome (“A familial syndrome of facial and skeletal anomalies associated with genital abnormality in the male and normal genitals in the female: Another cause of male pseudohermaphroditism” 1965). Occurring in 1/2000–4000 live births and 1/1000 fetuses, (Blagojevic et al., 2021; Maisenbacher et al., 2017) this syndrome is the most known recurrent microdeletion. Carriers of this microdeletion exhibit a broad range of phenotypic variability (Oskarsdóttir et al., 2004) spanning from craniofacial anomalies (40%), conotruncal defects of the heart (70–80%), hypoparathyroidism/hypocalcemia (20–60%), and subtle dysmorphic facial features, accompanied by a range of executive function (EF) deficits including visuospatial difficulties and weaknesses in abstract reasoning (Antshel et al., 2005; Cascella & Muzio, 2015; Jolin et al., 2009; Jonas et al., 2014; Stephenson et al., 2015). In addition, psychiatric comorbidities such as ADHD and anxiety in childhood and a high risk for the development of psychosis in adulthood are observed. The spectrum of Intellectual functioning widely varies, ranging from severe to average intellectual functioning, with the majority of individuals with 22qDS having an IQ that falls in the borderline range (IQ from 70 to 84), and about 1/3 have mild to moderate intellectual disability (ID) with the cognitive decline observed in both adolescents and adults and preceding the onset of psychosis (Antshel et al., 2016; Duijff et al., 2012; Fiksinski et al., 2022; Swillen & McDonald-McGinn, 2015).

In adulthood, 20–30% of individuals with 22qDS develop a psychotic disorder, particularly schizophrenia spectrum disorders (Bassett & Chow, 2008; Schneider et al., 2014; Van et al., 2017). Other psychiatric conditions associated with 22qDS include autism spectrum disabilities, ADHD, mood, and anxiety disorders. In addition, many hematological abnormalities, including thrombocytopenia, a low blood platelet count, with increased average size and volume of platelets, are quite frequent and likely related to immunodeficiency, a common finding in 40–95% of individuals with 22qDS (Akar & Adekile, 2007; Rosa et al., 2011).

Longitudinal studies in 22qDS provide insights into potential demographic, cognitive, clinical, and neuroimaging predictors of clinical outcomes (Tang & Gur, 2018). A recent case–control association study involving 1053 individuals with 22qDS, and controls, reveals the modifiers i.e., *Crkl* of the conotruncal heart defects (Zhao et al., 2020). A study including the assessment of 76 youths with 22qDS and 30 unaffected siblings, at three-time points, reported alterations in the cerebral surface morphology during late adolescence/early adulthood providing clinically relevant information about the psychiatric phenotype associated with the longitudinal trajectory of cortical surface morphology in the young population (Radoeva et al., 2017).

Metabolic and proteomic alterations are associated with various neurodevelopmental disorders, but limited research has been done in 22qDS and none have explored them longitudinally. A recent report performed plasma metabolomics and reported a shift from oxidative phosphorylation to glycolysis along with an increase in reductive carboxylation in individuals with 22qDS as compared to healthy controls (Napoli et al., 2015). Another study performed the proteomic and metabolomic brain profiling of the mouse model and reported changes in various molecular pathways associated with RNA transcription and chromatin remodeling along with an alteration in mitochondrial function, glycolysis/ gluconeogenesis, and lipid biosynthesis. They also observed changes in sphingomyelin, ceramide phosphoethanolamines, tyrosine derivates, carnitines, and pantothenic acid levels (Wesseling et al., 2017). A more recent study identified the metabolic signature for 22qDS in dried blood spots along with the associations of these metabolomic patterns with low intellectual functioning and ASD (Korteling et al., 2022).

Several studies reported on the link between specific elements of the complement cascade, key components of the immune system, and psychiatric disorders (Druart & Magueresse 2019; Westacott et al., 2022). The complement cascade participates in the development of synapse pruning (Bennett & Molofsky 2019) and, specifically, in pathological synapse removal in disorders that are associated with psychiatric manifestations (Westacott et al., 2022). In addition, increased levels of the complement components C3a and C5a, have been reported in patients with bipolar disorders (Yang et al., 2018). Interestingly, several genes mapping within the deleted region of 22qDS have been associated with complications of this immune system (TBX-1, DGCR8, and CRKL), psychosis (COMT, PRODH, *GNB1L*, *SEP5,* and *GP1BB* (Giacomelli et al., 2016; Jeker et al., 2013; Jerome & Papaioannou, 2001), congenital malformations and with other clinical phenotypes (Bassett et al., 2011; McDonald-McGinn et al., 2015; Meechan et al., 2015; Morrow et al., 2018; Morsheimer et al., 2017; Sullivan, 2019; Zemble et al., 2010; Zinkstok et al., 2019). However, their haploinsufficiency by itself cannot account for the heterogeneity in the severity and penetrance of the clinical involvements among those affected. Thus, not a single causal gene within the deleted region, which could explain the high prevalence of the observed 22qDS phenotypes, has been yet identified. Moreover, although psychiatric disorders considerably impact human health, no reliable prognostic biomarkers are currently available.

In this longitudinal cohort-sequential study we investigated the metabolic biomarkers and examined how complex proteomic pathways change over time within participants affected with 22qDS, and also as compared to HC. A decreased level of complex lipid metabolites, including arachidonic acid and taurine, was observed in the 22qDS as compared to the HC group. Further, we observed changes in the expression levels of genes of the complement and coagulation cascade, including *C3, C4B, SERPINA1*, and *SERPING1* which play a central role in the development of psychiatric symptoms. We also compared the metabolic and proteomic profiles of the participants with 22qDS with and without medical, and psychiatric conditions to detect the changes at various time points. We observed a large number of metabolites and proteins whose expression level was statistically significant among all these groups, but the significance went away after correction.

## Materials and methods

### Study participants

As part of a longitudinal study, participants were recruited across the United States. The study was carried out in accordance with the Institutional Review Board (IRB) at the University of California, Davis with written informed consent obtained from all participants in accordance with the Declaration of Helsinki. The majority of the participants with 22qDS exhibited the hemizygous 3 Mb deletion region of chromosome 22 spanning ~ 30 to 40 genes with only 5–10% of them having the 1.5 Mb deletion (Sellier et al., 2014).

The deletion size was characterized by deletion endpoints analysis using our developed ddPCR approach as previously described (Hwang et al., 2014). Two groups of participants were included in this study: 22qDS, and healthy controls (HC). They were matched by gender and age; the biological specimens were derived from participants at both baselines, V1 (22qDS, n = 16; HC, n = 14) and from 22qDS (n = 16) with various conditions including psychiatric, hematological, medical conditions and ASD at the follow-up visit (V2). Comorbid conditions were abstracted from medical screening forms and clinician interviews of parents regarding prior diagnoses. “Medical” involvement included the presence of any of the following conditions: congenital heart disease, endocrine dysfunction, renal abnormalities, immune dysregulation, and hematological conditions (present vs. absent), including any of the following: thrombocytopenia, anemia, and/or neutropenia. This was then rated as severe, mild, or absent. Autism (present vs. absent) was determined by parent reports of previous diagnosis and “other psychiatric” conditions including ADHD or anxiety.

### Sample handling and preparation

Blood samples were collected between 9:00 and 11:00 am under fasting conditions, in a purple-top tube (EDTA) for plasma collection. Within 2 h. The plasma was isolated by centrifugation for 10 min at 1000×*g* and stored at – 80 °C until processing. Samples were randomized and prepared using an automated liquid handling system. Multiple isotopically labeled recovery standards were added prior to the first preparation step for quality control purposes (QC). Samples were buffered with ammonium bicarbonate, chelated with EDTA, and denatured with an organic solvent. The protein lysate samples were digested with trypsin to generate LC–MS amenable peptides. To remove the undigested matrix, the digest was precipitated with additional organic solvents followed by centrifugation. The resultant supernatant extract was transferred and stored at 4 °C until further analysis.

### Liquid chromatography mass spectrometry (LC–MS)

Samples were analyzed using a mixed-mode chromatography system coupled to a high-resolution mass spectrometer via electrospray ionization (Thermo Scientific Q Exactive Plus). The injected sample preparation was loaded onto a reverse phase column (Waters CSH C18) and the flowthrough was diluted in-line for loading onto a HILIC column (Waters BEH Amide). The columns were eluted sequentially using gradients composed of water, acetonitrile, and isopropanol modified with mobile phase additives including formic acid and ammonium acetate. For quantification, the data was acquired using MS1 scans in both positive and negative ion modes, and for identifying the data was acquired using data dependent MS2 scans with dynamic exclusion. The mass analyzer was operated at 35,000 to 70,000 mass resolution, covering a scan range of approximately 80 to 1200 m/z. The LC–MS data was recorded in Thermo RAW format which was later converted to open ML format prior to data processing. For identification, pooled samples were analyzed using the data-dependent acquisition of MS2 spectra separately for peptides and for metabolite/lipid extracts.

### Data processing and analysis

#### Identification

Ions from the data dependent MS2 data on pooled samples were identified by comparison to MS2 libraries composed of experimental and theoretical spectra. Species-specific protein databases were theoretically digested and fragmented for predicted MS2 ions (MSFragger FDR < 0.01). Theoretical lipid MS2 spectra were generated from fatty acyl chains and head groups (LipiDex, forward dot product > 0.5 & reverse dot product > 0.7). Metabolite and small molecule MS2 spectra were from experimentally acquired spectral libraries (Compound Discoverer, match score > 0.8).

#### Relative quantification

The raw MS1 data were calibrated and quantified based on the retention times and m/z’s of identified ions. Label-free quantification with identifications matched between runs (LFQ-MBR) was used to extract peak intensities sums from analytes identified from the data-dependent MS2 acquisition of pooled metabolite extracts and fractionated peptide DDA runs. Ions were filtered for minimum coverage across experimental samples (coverage > 50%), and absence in QC water samples. Outlier samples were rejected based on the excess correlation-based distance from other sample profiles. Analyte profiles across samples were statistically corrected for run order effects by retaining residuals after regressing log10 intensity on run order in using linear models. Multiple analyte forms of biochemicals were condensed into molecule-level relative quantification by averaging, e.g., multiple peptides and charge states were condensed into single parent protein, multiple adduct forms of lipids were condensed into isomeric lipid species, etc.

#### Statistical analysis

Statistical correlations between biochemicals and experimental variables (e.g., treatment/group, age, sex, race, etc.) were tested using crude correlations and multiple linear/logistic regression. Multiple testing was addressed using false discovery rate (FDR) correction of nominal significance (P value). Differential associations were visualized using volcano plots and global data patterns are visualized with dimensionality reduction plots (hierarchical clustering, PCA, & UMAP). Differential metabolite/protein/small molecule expression analyses were conducted using the R package limma, version 3.46.0, running in R version 4.0.5P values for age were from a Wilcoxon rank-sum test and P values for gender are from Fisher’s Exact Test.

### Maintenance and quality control

LC–MS instrumentation was cleaned, calibrated, and tuned on a regular basis according to manufacturer recommendations. Instrument performance was monitored using a QC digest standard for an expected number of identifications. Pooled quality control samples were interspersed within each batch to assess data quality and to correct for technical effects such as batch and run order effects. Water and blank preparations were also interspersed to assess carryover and contamination. A cocktail of isotopically labeled internal analytical standards was added to each sample to allow monitoring of instrument performance, and to assess recovery and precision of quantification. Experimental samples were randomized within and across batches with technical sample preparation replicates and QC samples interspersed within each batch. After relative quantification, the experimental sample’s global biochemical profiles were expected to be highly correlated (r > 90% and 95% for biological and technical replicates respectively). Suitable endogenous protein subunits were compared for stoichiometric agreement, e.g., subunits A and B are expected to be highly correlated with each other (r > 90%).

### Pathway analysis

Pathway analysis was performed using the Epistemic AI platform (epistemic.ai) using the results obtained by mass spectrometry. Only pathways with a P value and FDR of less than 0.001 were considered.

## Results

**Fig. 1:**
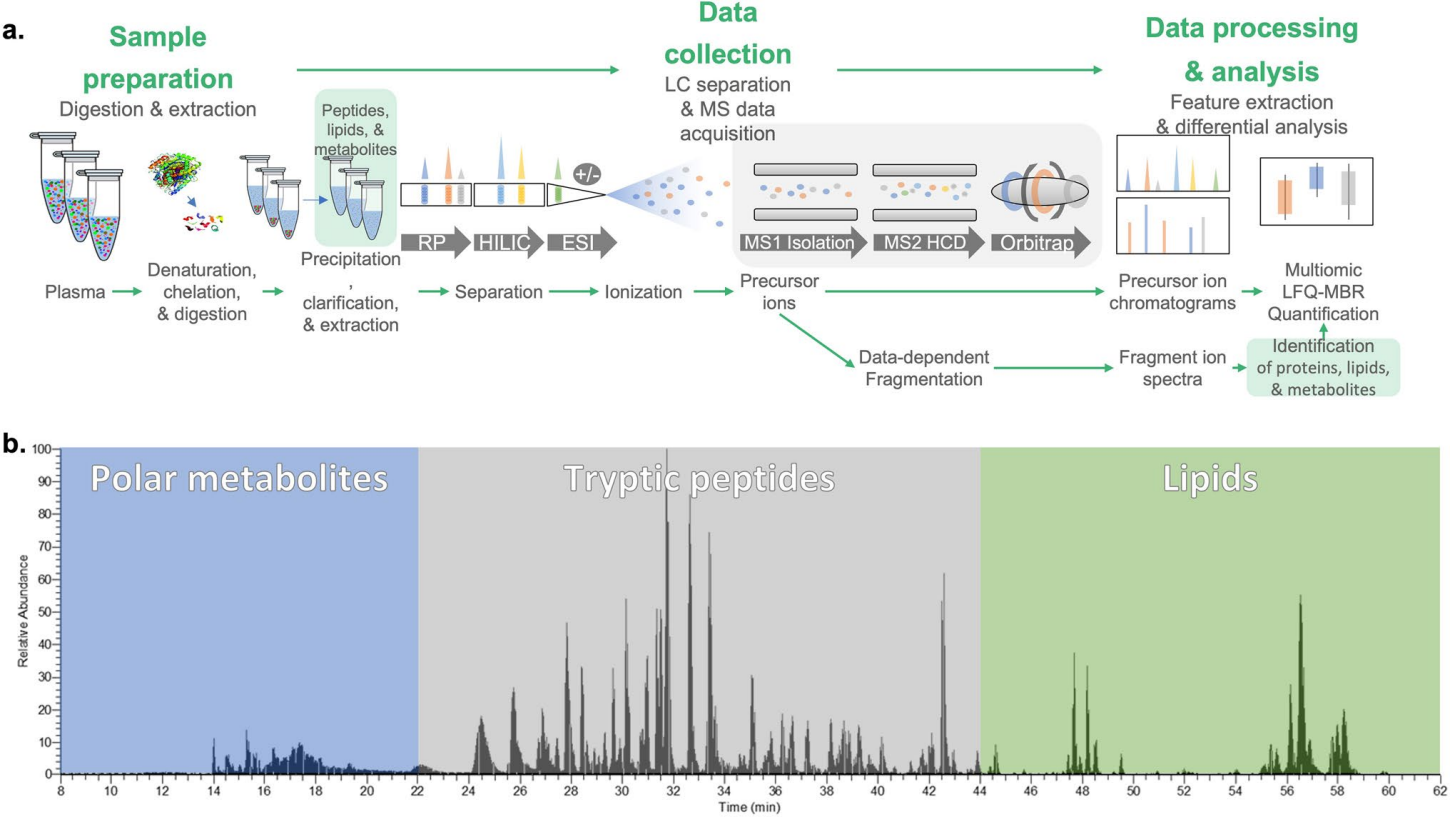
**a** Multiomics analysis with the Omni-MS workflow. Plasma samples are randomized and aliquoted in for preparation, undergoing solvent denaturation, metal chelation, and trypsin digestion. The undigested debris is solvent precipitated and clarified with centrifugation. The multiomic extract is injected into the LC–MS instrument, separated using reverse phase and HILIC chromatography, ionized by ESI, and data collected by high resolution MS1 scans and data-dependent MS2 scans. The ddMS2 data is used to determine identifications, which are in turn matched between runs to generate label-free quantification between samples. **b** Multiomic LC–MS separation. The figure shows the total ion chromatogram (TIC) visualization for a representative plasma sample preparation containing polar metabolites, tryptic peptides, and lipids after analysis via the RP-HILIC LC–MS system. The y-axis shows the relative total ion intensity of eluting components (metabolites, tryptic peptides, and lipids) as a function of retention time (x-axis)

In this study, we characterized and compared a global metabolomic and proteomic profiling in the 22qDS and HC groups, and within the 22qDS group, to identify novel differentially expressed metabolites and proteins using the multi-omics technology (Fig. 1a) with LC–MS separation (Fig. 1b).

### Demographics

Metabolomic and proteomic profiling was obtained from 30 participants including males (n = 10 22qDS, n = 6 HC) and females (n = 6 22qDS, n = 8 HC). Gender and age were as reported in Table 1. There were no gender differences (P = 0.2069) between the two groups and participants within the 22qDS group (n = 16) with various conditions including psychiatric, medical hematological, and ASD were assessed at various time points.

**Table 1:** Participant demographics

	TD (n = 14)	22q11.2 DS (n = 16)	P value
Age			0.491
N	14	16	
Mean (SD)	12.7 (3.1)	11.9 (3.3)	
Median (range)	13.5 (7–17)	11.5 (7–17)	
Gender			0.464
F	8 (57.1%)	6 (37.5%)	
M	6 (42.9%)	10 (62.5%)	

### Differential metabolite levels between the 22qDS and the HC groups

**Fig. 2:**
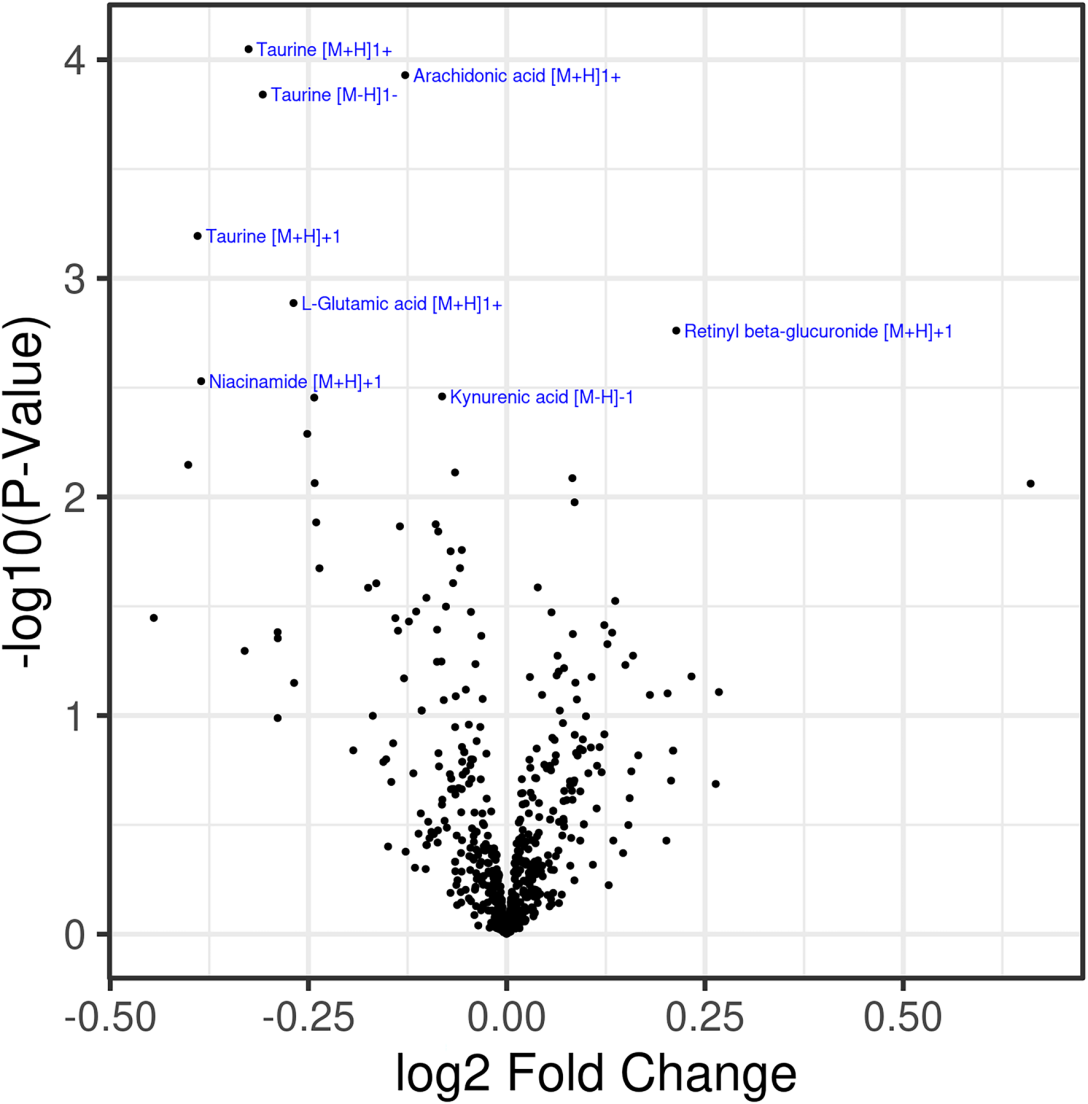
Differential metabolite levels between 22q11.2DS and the groups. Volcano plot of differential expression results comparing metabolite expression in the 22q11.2DS and the TD subjects at baseline. The x-axis shows the log2 fold change for 22q11.2DS/TD and the y-axis shows − log10 (raw P value). The name of the 8 most significant metabolites is indicated in blue and these plots are generated using R software

**Fig. 3:**
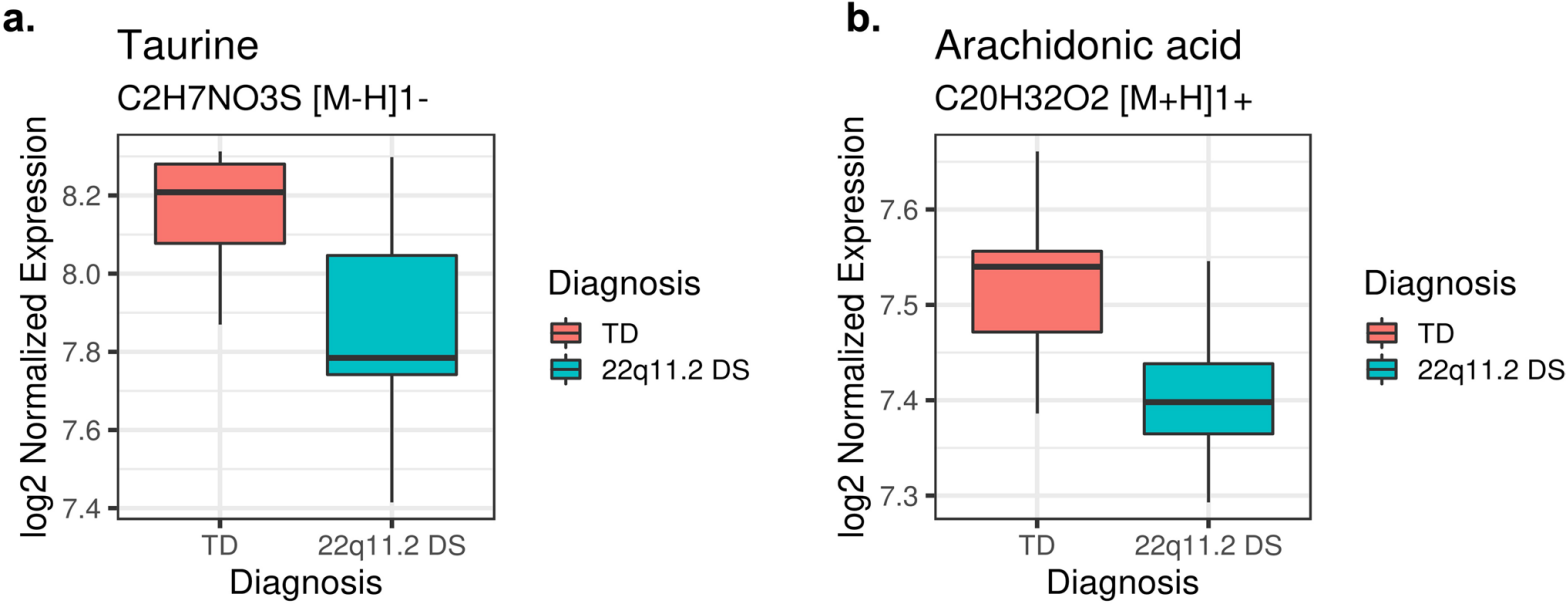
Taurine and arachidonic acid levels between TD and 22q11.2DS groups. Box plots showing decreased levels of taurine (**a**) and of arachidonic acid (**b**) in 22q11.2DS as compared to TD. The heavy line in each box represents the median, the lower and upper box edges represent the 25th and 75th percentiles, respectively, and the lower and upper whiskers represent the smallest and largest observations, respectively

To identify metabolic biomarkers potentially associated with the development and progression of 22qDS, we compared the untargeted metabolic profile of 22qDS to the HC group. Within the 628 detected metabolites (Fig. 2), 53 showed statistically significant changes in level (P < 0.05), including several omega-3-polyunsaturated fatty acids, and 3 were statistically significant after FDR adjustment (adjusted P < 0.05). Among them, taurine (Adjp = 0.04036; Fig. 3a) and Arachidonic acid (Adjp = 0.04036; Fig. 3b) showed a significantly decreased level in 22qDS compared to HC even after Benjamini–Hochberg false discovery rate adjustment.

### Differential protein levels between the 22qDS and the HC groups

**Fig. 4:**
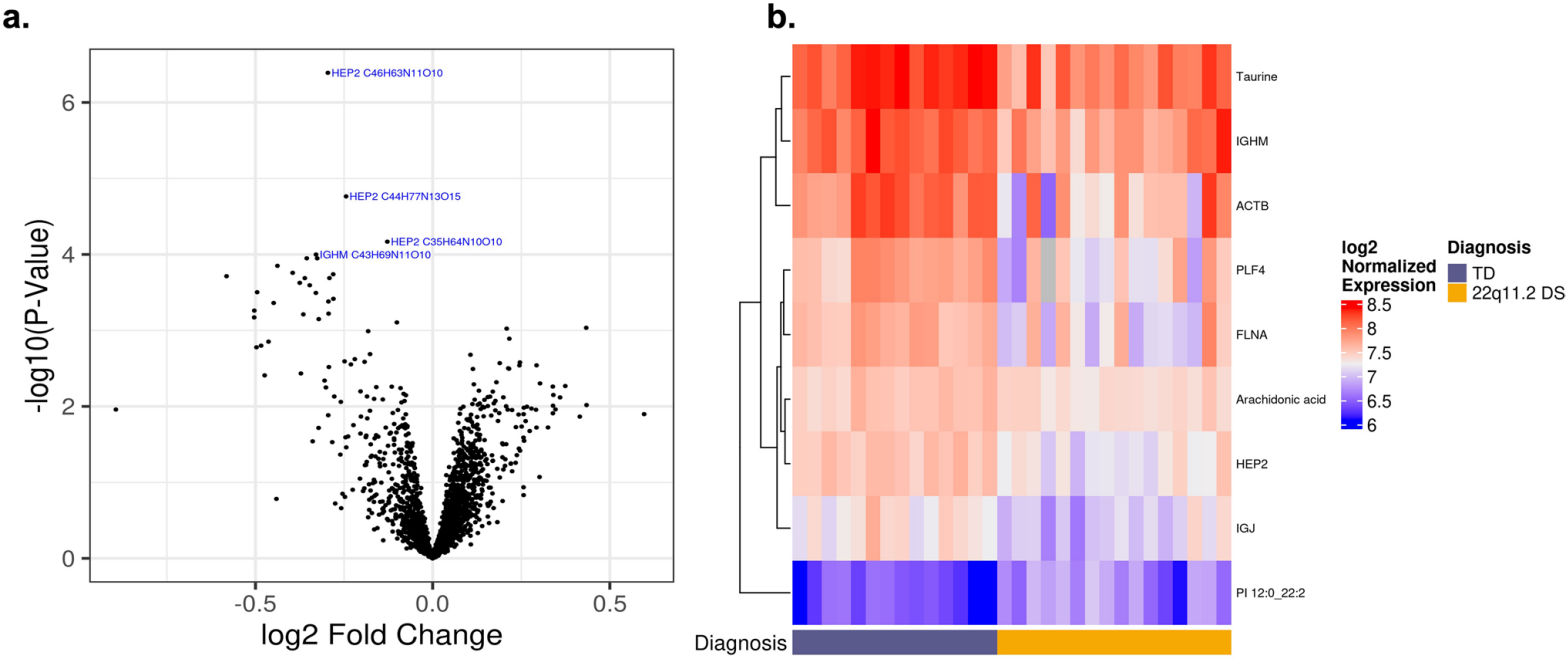
Differential proteins levels between 22q11.2DS and the TD groups. **a** The volcano plot was generated using R software. Volcano plot of differential expression results comparing protein expression in 22q11.2DS and TD subjects at baseline. The x-axis shows the log2 fold change for 22q11.2DS /TD and the y-axis shows − log10 (raw P value). The name and formula of the 5 most significantly proteins are shown in blue. **b** Heatmap of proteins, lipids, and metabolites that are differentially expressed (adjusted P < 0.05) between 22q11.2DS and TD subjects at baseline. Rows are sorted based on the hierarchical clustering dendrogram shown on the left-hand side and columns are sorted by subject diagnosis

From the untargeted proteomic profiling, we identified 274 total proteins (Fig. 4a) out of which, 16 showed significant changes in expression (*P* < 0.05) between the 22qDS and the HC groups, suggesting their role in the development of comorbid conditions (Fig. 4b; Table 2). 14 of these were significant following FDR adjustment (adjusted P < 0.05). Interestingly, among these proteins, there were the Heparin cofactor 2, Immunoglobulin heavy constant mu, the platelet factor 4, Filamin-A and Actin, cytoplasmic 1, Thymosin beta-4 which locus maps within the deleted region of chromosome 22 and found to be strongly associated with psychosis.

**Table 2:** Differential protein expression levels between the 22q11.2DS and the TD groups

Sr #	Name	Description	logFC	AveExpr	P value	adj.P.Val	Formula	Uniprot	Peptide	Ion	Type
1	HEP2/ SERPIND1	Heparin cofactor 2	− 0.29567	7.38517	0	0.00128	C46H63N11O10	P05546	FAFNLYR	[M + 2H]2+	protein
2	HEP2/ SERPIND1	Heparin cofactor 2	− 0.24434	7.6289	0.00002	0.02712	C44H77N13O15	P05546	TLEAQLTPR	[M + 2H]2+	protein
3	HEP2/ SERPIND1	Heparin cofactor 2	− 0.12795	7.60783	0.00007	0.04036	C35H64N10O10	P05546	LNILNAK	[M + 2H]2+	protein
4	IGHM	Immunoglobulin heavy constant mu	− 0.32915	7.96117	0.0001	0.04036	C43H69N11O10	P01871	n (Lim et al., 2019) VSVFVPPR	[M + 2H]2+	protein
5	IGHM	Immunoglobulin heavy constant mu	− 0.35531	8.4965	0.00011	0.04036	C66H112N20O27	P01871	QVGSGVTTDQVQAEAK	[M + 2H]2+	protein
6	IGHM	Immunoglobulin heavy constant mu	− 0.32583	8.09937	0.00011	0.04036	C36H58N10O9	P01871	GFPSVLR	[M + 1H]1+	Protein
7	PLF4	Platelet factor 4	− 0.43806	7.49934	0.00014	0.04036	C47H82N12O14	P02776	HITSLEVIK	[M + 2H]2+	Protein
8	FLNA	Filamin-A	− 0.39502	7.43397	0.00017	0.04036	C62H99N17O22	P21333	ANLPQSFQVDTSK	[M + 2H]2+	Protein
9	IGHM	Immunoglobulin heavy constant mu	− 0.28069	8.6498	0.00018	0.04036	C66H111N15O17	P01871	NVPLPVIAELPPK	[M + 2H]2+	Protein
10	ACTB	Actin, cytoplasmic 1	− 0.58166	7.73957	0.00019	0.04036	C142H223N37O44S1	P60709	TTGIVMDSGDGVTHTVPIYEGYALPHAILR	[M + 4H]4+	Protein
11	IGJ	Immunoglobulin J chain	− 0.29129	7.1815	0.0002	0.04036	C55H88N16O26	P01591	SSEDPNEDIVER	[M + 2H]2+	Protein
12	IGHM	Immunoglobulin heavy constant mu	− 0.36074	7.46153	0.00021	0.04036	C70H109N19O22S1	P01871	YVTSAPMPEPQAPGR	[M + 2H]2+	Protein
13	IGHM	Immunoglobulin heavy constant mu	− 0.37507	7.70203	0.00024	0.04385	C82H124N16O19	P01871	VFAIPPSFASIFLTK	[M + 2H]2+	Protein
14	IGHM	Immunoglobulin heavy constant mu	− 0.34688	8.07107	0.00025	0.04452	C58H96N14O18	P01871	YAATSQVLLPSK	[M + 2H]2+	Protein
15	TYB4	Thymosin beta-4	− 0.49529	7.4072	0.00031	0.05055	C65H109N17O24	P62328	NPLPSKETIEQEK	[M + 3H]3+	Protein
16	HEP2/ SERPIND1	Heparin cofactor 2	− 0.32954	7.35303	0.00032	0.05055	C48H78N12O16	P05546	SVNDLYIQK		

### Enrichment analysis identifies altered pathways between the 22q11.2DS and the TD groups

**Fig. 5:**
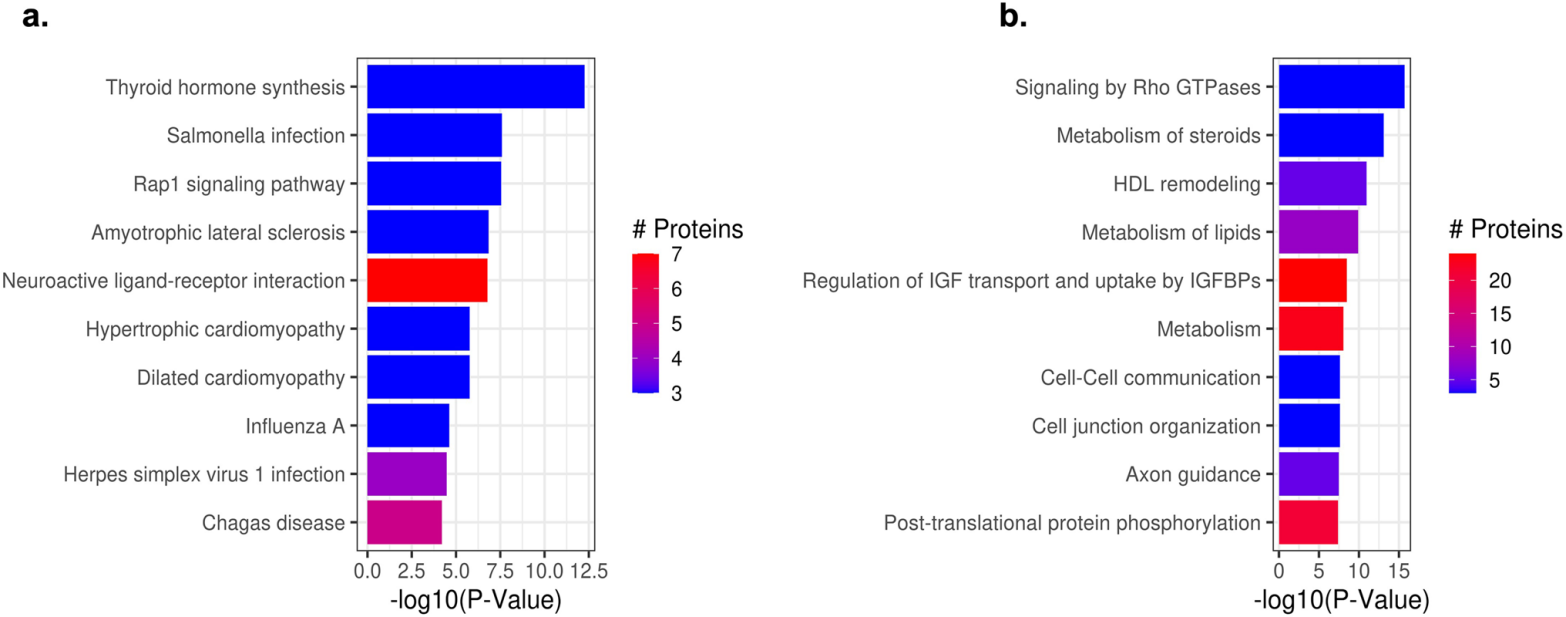
Altered protein pathways observed between the 22q11.2DS and the TD groups. Barplot of top 10 most significantly enriched KEGG pathways (**a**) and Reactome pathways, (**b**) from enrichment analysis of differential expression of proteins between 22q and TD at baseline. The length of the bars shows − log10 (P value) from the enrichment analysis and the color of the bars shows the number of proteins included in the DE analysis (regardless of significance) in the indicated pathway

**Fig. 6:**
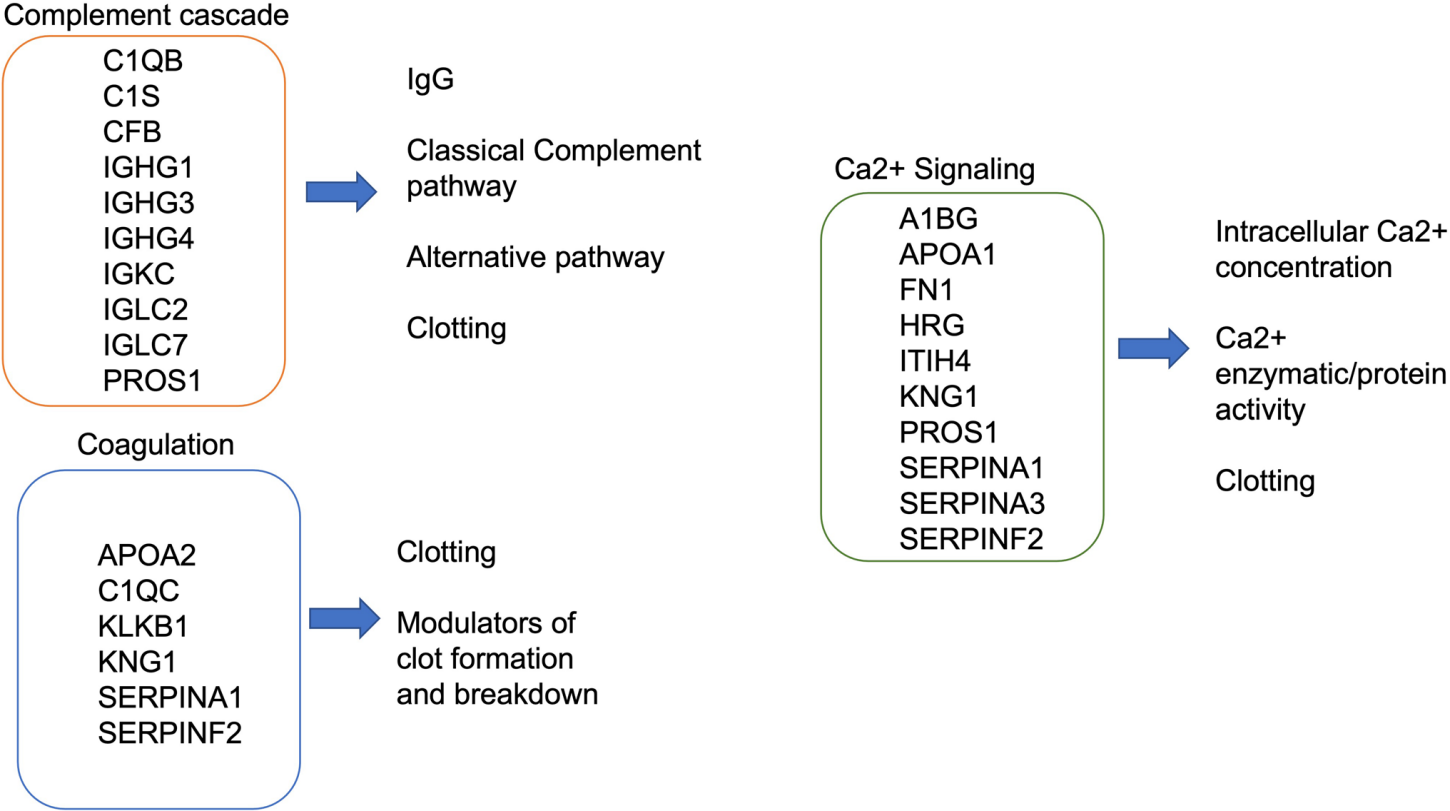
Altered pathways between 22q11.2DS and TD including representative genes in the complement cascade, coagulation and Ca2 + signaling pathways, other psychiatric and autism altered protein datasets, and some of their known. The figure shows selected enriched genes identified by multiple pathway databases, including Reactome, Bioplanet and Wikipathways. Enrichment analysis was generated using the Epistemic AI platform

The KEGG (Fig. 5a) and Reactome (Fig. 5b) enrichment analysis between 22q11.2DS and TD at baseline showed significant differential protein expression levels of 70 pathways. These included gene expressions, the immune system, the Rap1 signaling pathway, the PI3K-Akt signaling pathway, the intrinsic pathway of fibrin clot formation, the pathway involved in the initial triggering of complement, and those involved in the activation of C3 and C5 (Fig. 6) and Interleukin-4 and Interleukin-13 signaling (Table 3).

**Table 3:** Significantly enriched protein pathways for 22q11.2DS vs TD at baseline

Sr #	Pathway code	Pathway name	P value	Annotated
1	R-HSA-194315	Homo sapiens: signaling by Rho GTPases	0	3
2	R-HSA-8957322	Homo sapiens: metabolism of steroids	0	3
3	hsa04918	Thyroid hormone synthesis—homo sapiens (human)	0	3
4	R-HSA-8964058	Homo sapiens: HDL remodeling	0	5
5	R-HSA-556833	Homo sapiens: metabolism of lipids	0	8
6	R-HSA-381426	Homo sapiens: regulation of insulin-like growth factor (IGF) transport and uptake by insulin-like growth factor binding proteins (IGFBPs)	0	24
7	R-HSA-1430728	homo sapiens: metabolism	0	23
8	R-HSA-1500931	Homo sapiens: cell–cell communication	0	3
9	R-HSA-446728	Homo sapiens: cell junction organization	0	3
10	hsa05132	Salmonella infection—homo sapiens (human)	0	3
11	hsa04015	Rap1 signaling pathway—homo sapiens (human)	0	3
12	R-HSA-422475	Homo sapiens: axon guidance	0	5
13	R-HSA-8957275	Homo sapiens: post-translational protein phosphorylation	0	21
14	R-HSA-9675108	Homo sapiens: nervous system development	0	7
15	R-HSA-392499	Homo sapiens: metabolism of proteins	0.0000001	41
16	R-HSA-1266738	Homo sapiens: developmental biology	0.0000001	9
17	hsa05014	Amyotrophic lateral sclerosis—homo sapiens (human)	0.0000001	3
18	hsa04080	Neuroactive ligand-receptor interaction—homo sapiens (human)	0.0000002	7
19	R-HSA-8963899	Homo sapiens: plasma lipoprotein remodeling	0.0000008	8
20	R-HSA-168249	Homo sapiens: innate immune system	0.0000013	53
21	hsa05410	Hypertrophic cardiomyopathy—homo sapiens (human)	0.0000017	3
22	hsa05414	Dilated cardiomyopathy—homo sapiens (human)	0.0000017	3
23	R-HSA-8856828	Homo sapiens: clathrin-mediated endocytosis	0.0000056	3
24	R-HSA-76002	Homo sapiens: platelet activation, signaling and aggregation	0.0000062	37
25	R-HSA-168256	Homo sapiens: immune system	0.0000092	61
26	R-HSA-114608	Homo sapiens: platelet degranulation	0.0000113	36
27	R-HSA-76005	Homo sapiens: response to elevated platelet cytosolic Ca2 +	0.0000113	36
28	hsa05164	Influenza A—homo sapiens (human)	0.0000235	3
29	R-HSA-373076	Homo sapiens: class A/1 (rhodopsin-like receptors)	0.0000329	7
30	R-HSA-375276	Homo sapiens: peptide ligand-binding receptors	0.0000329	7
31	R-HSA-500792	Homo sapiens: GPCR ligand binding	0.0000329	7
32	hsa05168	Herpes simplex virus 1 infection—homo sapiens (human)	0.0000331	4
33	R-HSA-597592	Homo sapiens: post-translational protein modification	0.0000562	33
34	hsa05142	Chagas disease—homo sapiens (human)	0.000064	5
35	R-HSA-372790	Homo sapiens: signaling by GPCR	0.0000708	17
36	R-HSA-388396	Homo sapiens: GPCR downstream signaling	0.0000708	17
37	R-HSA-418594	Homo sapiens: G alpha (i) signaling events	0.0001343	14
38	R-HSA-74160	Homo sapiens: gene expression (transcription)	0.0001737	10
39	R-HSA-140837	Homo sapiens: intrinsic pathway of fibrin clot formation	0.0001864	13
40	R-HSA-166663	Homo sapiens: initial triggering of complement	0.0002342	13
41	R-HSA-174577	Homo sapiens: activation of C3 and C5	0.0002704	7
42	R-HSA-140877	Homo sapiens: formation of fibrin clot (clotting cascade)	0.0003324	18
43	R-HSA-1237044	Homo sapiens: erythrocytes take up carbon dioxide and release oxygen	0.000406	3
44	R-HSA-1247673	Homo sapiens: erythrocytes take up oxygen and release carbon dioxide	0.000406	3
45	R-HSA-1480926	Homo sapiens: O2/CO2 exchange in erythrocytes	0.000406	3
46	hsa05203	Viral carcinogenesis—homo sapiens (human)	0.0004301	3
47	R-HSA-446203	Homo sapiens: asparagine N-linked glycosylation	0.0004441	3
48	R-HSA-8963898	Homo sapiens: plasma lipoprotein assembly	0.0005163	8
49	R-HSA-1643685	Homo sapiens: disease	0.0005245	27
50	R-HSA-3000171	Homo sapiens: non-integrin membrane-ECM interactions	0.0005636	3
51	hsa05144	Malaria—homo sapiens (human)	0.0006717	4
52	hsa05200	Pathways in cancer—homo sapiens (human)	0.0006815	8
53	R-HSA-75205	Homo sapiens: dissolution of Fibrin Clot	0.0024678	3
54	hsa04611	Platelet activation—homo sapiens (human)	0.00393	7
55	R-HSA-3000178	Homo sapiens: ECM proteoglycans	0.0039526	3
56	R-HSA-1566948	Homo sapiens: elastic fiber formation	0.0044953	3
57	R-HSA-2129379	Homo sapiens: molecules associated with elastic fibers	0.0044953	3
58	hsa04060	Cytokine-cytokine receptor interaction—homo sapiens (human)	0.0074959	3
59	hsa04061	Viral protein interaction with cytokine and cytokine receptor—homo sapiens (human)	0.0074959	3
60	hsa04151	PI3K-Akt signaling pathway—homo sapiens (human)	0.0080034	6
61	R-HSA-1474228	Homo sapiens: degradation of the extracellular matrix	0.0096078	5
62	R-HSA-1280218	Homo sapiens: adaptive Immune System	0.0102135	3
63	R-HSA-449147	Homo sapiens: signaling by Interleukins	0.011517	5
64	hsa04512	ECM-receptor interaction—homo sapiens (human)	0.0155588	6
65	R-HSA-6785807	Homo sapiens: interleukin-4 and interleukin-13 signaling	0.0169009	3
66	hsa05165	Human papillomavirus infection—homo sapiens (human)	0.0193635	5
67	hsa04510	Focal adhesion—Homo sapiens (human)	0.0291135	7
68	R-HSA-5619115	Homo sapiens: disorders of transmembrane transporters	0.0311366	3
69	R-HSA-416476	Homo sapiens: G alpha (q) signaling events	0.0384096	3
70	R-HSA-9651496	Homo sapiens: defects of contact activation system (CAS) and kallikrein/kinin system (KKS)	0.0474929	7
71	R-HSA-9671793	Homo sapiens: diseases of hemostasis	0.0474929	7

### Differential metabolite and protein expression levels within the 22q11.2DS group at different time points

We assessed the differentially expressed metabolites and proteins within the 22q11.2DS groups as follows: in the 5 22q11.2DS subjects with Attenuated Positive Symptom Prodromal Syndrome (APS) on a scale of 1 vs. 0 at any timepoint, we observed 60 metabolites and 117 proteins differentially expressed (P > 0.05; Supplementary Table 3a, b, 0 significant features following FDR adjustment). In the 2 22q11.2DS subjects with hematological issues on a scale of 1 or 2 vs. 0 at any timepoint, we found 33 metabolites and 184 proteins differentially expressed [P > 0.05; Supplementary Table 4a, 4b, 0 significant features following FDR adjustment]. In 16 22q11.2DS subjects with medical issues at any timepoint, on a scale of 2 vs 0 or 1, we found 38 metabolites and 51 proteins differentially expressed (P > 0.05; Supplementary Table 5a, b, 0 significant features following FDR adjustment). In the 12 22q11.2DS subjects with other psychiatric issues at any timepoint, on a scale of 1 vs 0, we found 27 metabolites and 199 proteins differentially expressed (P > 0.05; Supplementary Table 6a, b, 0 significant features following FDR adjustment), and lastly, in the 3 22q11.2DS subjects with ASD on a scale of 1 vs 0 we found that 8 metabolites and 122 proteins were differentially expressed (P > 0.05; Supplementary Table 7a, b, 0 significant features following FDR adjustment). However, all of the metabolites and the proteins lost significance after Benjamini-Hochberg’s false discovery rate correction.

### Differential pathway involvement in 22q11.2DS participants with psychiatric symptoms

We observed changes in multiple pathways involving the complement system, coagulation, and altered calcium signaling in blood, especially in 22q11.2DS participants presenting with ASD and psychiatric symptoms. Among the proteins associated with the differences in the ASD group, there were pathway enrichments in Ca2 + signaling in platelets from Reactome (P < 0.001), and platelet alpha granule from Cellular Components (P < − 0.001). In association with other psychiatric disorders, complement cascade from Bioplanet (P < 0.001), Complement and Coagulation Cascades from Wikipathways, (P < 0.001), and Changes in Ca2 + Related platelet signaling Reactome, (P < 0.001) (Supplementary Table 6). Specifically, some proteins of interest whose expression was altered between the 22q11.2DS and the TD groups were A1AT, A1BG, APOA1, HRG, KMG1, SERPINA3, and IGHG1.

## Discussion

These findings expand upon an earlier, more limited study of metabolic and proteomic profiling of patients with a definitive diagnosis of 22qDS (Korteling et al., 2022) and in the prefrontal cortex (PFC) and hippocampal (HPC) tissue of the murine model, (Wesseling et al., 2017) confirming the involvement of pathways previously identified but now expanded (Table 3) as well as the involvement and alterations of several other pathways. To our knowledge, this is the first report of the longitudinal metabolic and proteomic profiling and on the identification of unique biomarkers that might in the future be used for early diagnosis, development, and the progression of 22qDS (Fig. 4 and Table 2).

22qDS is characterized by a highly variable psychiatric phenotype across individuals, which includes anxiety, ADHD, and mood instability. A recent study found a DSM IV diagnosis rate of 76% in 5–12-year-olds, mainly represented by Disruptive behavior (55%), ADHD, and anxiety disorders (49%) (Green et al., 2009).

In this study, we reported two metabolites, taurine, and arachidonic acid as potential molecular determinants of psychiatric dysfunction. Taurine, the most abundant metabolite in the central nervous system, is a modulator of inhibitory neurotransmission and mediates this activity by binding to GABAA, GABAB, and glycine receptors. It is one of the most abundant and essential free amino acid with diverse cytoprotective activity, which plays a role in energy metabolism and shown to be relevant for brain development by decreasing ER stress and antagonizing neurotransmitter receptors of GABA_A_, glycine and NMDA(Furukawa et al., 2014; Tyson et al., 1989). Expression levels are variable across species, in different brain areas, and in developmental stages. Alterations in taurine homeostasis, with reduced brain taurine concentrations, have been reported in metabolic and neurodegenerative disorders and have a role in neural development and neurogenesis. Interestingly, taurine supplementation has been demonstrated to have protective effects in several disorders (Rafiee et al., 2022) by preventing toxicity in both neurons and astrocytes in vitro, as well as in animal models of neurological disorders (Jakaria et al., 2019). Further, administration of taurine has been shown to be beneficial in FXS /v) by linked to taurine’s GABAergic activity and in several neurodegenerative disorders inlcuding Parkinson disease and Alzheimer disease revealing a promise for the use of taurine as a therapeutic agent(Neuwirth et al., 2015; Ricciardi et al., 2015). The role and the positive effects of this taurine therapy in reducing the pathology and symptoms of several diseases affecting the CNS, the cardiovascular and the skeletal muscle systems, including stroke, neurodegenerative and neurodevelopmental disorders is reviewed by(Schaffer & Kim 2018). Further, a positive association between plasma taurine and neurodevelopment has been reported (Wharton et al., 2004) and, importantly, in a double-blind, randomized, placebo-controlled study, taurine improved psychopathology in patients with first-episode psychosis with significant improvements in positive symptoms and psychosocial functioning (Collin et al., 2020). Thus, our findings, of decreased plasma levels of taurine in 22qDS (Fig. 3a) point to it as a potential biochemical marker that could discriminate the patients with psychosis symptoms. They could also provide a better understanding of the pathophysiology of psychosis spectrum disorders and lead to the development of treatment in the early stages of the disease in 22qDS.

Arachidonic acid (AA omega-6; 20:4ω-6) is a long-chain polyunsaturated fatty acid (LCPUFA), the major n-6 LCPUFA, a structural component of the membrane phospholipid, specifically required for the formation of non-myelinated cell membranes in the CNS, and thus, important for neurodevelopment. Recently, several studies have shown its importance in cognitive function, attention, and memory (Ishikura et al., 2009; Kotani et al., 2006; Tokuda et al., 2014) and demonstrated lower levels in ASD (Meguid et al., 2008). In a few studies, positive effects of different arachidonic acid supplementation (Alshweki et al., 2015; Gould et al., 2013) with improved cognitive functions in the elderly (Tokuda et al., 2014), and improved impaired social interaction in children with ASD (Yui et al., 2012) have been demonstrated. In this study, we observed a decreased level of AA in 22qDS as compared to HC (Fig. 3b) suggesting its potential contribution to the pathogenesis of the disease.

In many diseases, including cancer, coronary atherosclerotic heart disease, psychosis, and depression, the protein product of *SERPIND1*, heparin cofactor II (HCII), plays an important role in preventing the onset of disseminated intravascular coagulation in several thromboses, including deep vein thrombosis (Hoogendoorn et al., 2004; Noda et al., 2002; Rein et al., 2011). The *SERPIND1* maps within the nested duplication region within the central 22qDS region between LCR22B and LCR22D suggest that its haploinsufficiency could be key and relevant to brain development in 22qDS (Woodward et al., 2019). Similarly, we observed a decreased expression of heparin cofactor II (HCII), mapping within the deleted region of 22qDS, in participants with 22qDS (Table 2) supporting its role in the pathogenesis of the disorder. The platelet factor 4 (PlF4) protein is associated with chronic idiopathic myelofibrosis and megakaryoblastic leukemia (AMGL). The origin of fibrosis in these disorders is a megakaryocytic proliferation in the bone marrow resulting in the inability to store platelet factor 4 (PlF4) (Palomera et al., 1989). Cardiovascular anomalies are common in 22qDS patients and could be triggered by altered functioning of the platelet factor 4 (PlF4), which we observed to be differentially expressed in the 22qDS as compared to HC (Table 2). Filamin A (*FLNA*) is an actin-binding protein, that plays pivotal roles in cell migration (Cunningham et al., 1992) and vascular development (Feng et al., 2006). Recent case control and a cohort study in the Chinese Han population support the genetic contribution of FLNA to hypertension (Liu et al., 2021). We found a lower expression of Filamin A in 22qDS (Table 2) suggesting its contribution to the observed psychiatric issues. β-actin protein (encoded by the *ACTB gene*) is a highly conserved cytoskeletal protein and participates in a variety of cell functions, such as the maintenance of cell shape, cell migration, division, growth, and signal transduction (Chen et al., 2016). Haploinsufficiency of ACTB due to the deletion of the gene was suggested as a reason for the clinical features observed in the patients with a 7p22.1 microdeletion (Palumbo et al., 2018). Interestingly, in this study, we observed a significant differential expression of the β-actin protein in 22qDS participants [Table 2]. Which suggests a role for some of the observed clinical features in these patients. The altered expression of all these proteins in the 22qDS suggests their potential contribution to the development of the disorder.

Immunodeficiency, present in 75% of patients with 22qDS (McDonald-McGinn et al., 2015) includes T‐cell dysfunction (Crowley et al., 2018), NK cell function deficiency (Zheng et al., 2015), and autoimmunity (Zemble et al., 2010). A significantly higher expression of inflammatory cytokines has been observed in individuals with 22qDS and, specifically, a significantly higher percentage of inflammatory Th1, Th17, and memory T-helper cells were reported in adults with 22qDS and a Th17 higher percentage in those with psychotic symptoms, which supports the involvement of these cells in the development of Schizophrenia Spectrum Disorders (SCZ) (Vergaelen et al., 2018). Immune-inflammatory alterations over different psychiatric disorders, including depression and stress, have been investigated and their correlation reported. Immune-inflammatory impairments including increased levels of pro-inflammatory cytokines have also been associated with psychiatric disorders, such as SCZ, depression, bipolar disorder (Khandaker et al., 2015; Miller et al., 2011), and autism spectrum disorders (Meltzer & Van de Water 2017; Siniscalco et al., 2018). However, the underlying mechanisms and the meaning of these associations are largely unknown.

Of relevance, there is a link between specific elements of the complement system, key components of the immune system, and psychiatric disorders (Druart & Le Magueresse 2019; Westacott et al., 2022). The complement system participates in the development of synapse pruning (Bennett & Molofsky 2019), and specifically, pathological synapse removal is associated with psychiatric manifestations (Westacott et al., 2022). Interestingly, increased levels of C3a and C5a components have been reported in patients with bipolar disorders (Yang et al., 2018). In this study, we observed impairment of the protein pathways involving the immune system, the activation of the C3 and C5 complement system (Fig. 6), and the autoimmune Interleukin-4 and Interleukin-13 signaling. These findings indicate that disturbance in these pathways is potentially associated with the development of the regulation of psychotic symptoms along with a correlation with clinical features. More specifically, in this study, we report changes in the complement system cascades and coagulation disruption in 22qDS patients with psychiatric symptoms [Supplementary Table 4b and 5b]. These data are consistent with clinical observations of complement changes in patients with psychiatric symptoms and 22qDS (Grinde et al., 2020). There are also clinical reports of patients with 22qDS being comorbid for clotting and coagulation disorders as well (Cohen et al., 2018). We also observed significant changes in proteins related to oxygen responses in those with psychiatric symptoms, especially ASD compared to HC [Supplementary Tables 4b and 5b], findings that are consistent with multiple case reports of hypoxemia in 22qDS patients, sometimes associated with the onset of CNS symptoms (Tonelli et al., 2007) and disruption of calcium being reported to disrupt synaptic plasticity in neurons in 22qDS murine model neurons (Devaraju & Zakharenko, 2017). Further, (Tonelli et al., 2007) reported a correlation between seizure onset and hypocalcemia in a 22qDS patient. Hypocalcemia is also often reported as a comorbidity of psychiatric conditions in 22qDS patients (Tang & Gur, 2018) and is associated with an increased risk of neurodevelopmental delays. Taken all together these data suggest a strong correlation between complement changes, coagulation, calcium signaling, and CNS symptoms in 22qDS. These proteins provide, therefore new areas for exploration in mechanisms and biomarkers.

Some limitations of this study include the chosen analytical method and non-causal study design. Here we employ an MS1-based label-free quantification using match between runs (LFQ-MBR) to transfer identifications from a library built using separate data-dependent MS2 acquisitions, an approach well established in proteomics, which, may, therefore, suffer from increased rates of false quantifications (Almeida et al., 2021; Geyer et al., 2016; Lim et al., 2019; Yu et al., 2021). However, in contrast to labeled (e.g., TMT) and LFQ-DIA approaches, LFQ-MBR is biomolecule-agnostic and is readily applied to not only peptides, but also lipids, metabolites, and other analytes, and an important part of this analysis. Although the longitudinal nature of this study enabled us to identify associations, however further work, importantly, in a larger cohort is needed to determine the causal influence of these analytes on phenotypes of interest.

## Conclusion

This is a longitudinal study, which identified metabolomic and proteomics alterations along with dysregulated protein pathways among individuals with 22qDS that represent potential predictors of medical and psychiatric diagnosis even before symptoms appear. They are suggested as biomarkers of prognosis and development for future pharmacological interventions. These alterations are not only providing us insights into the mechanisms involved in 22qDS but could also potentially help to understand the basic mechanisms leading to other neurodevelopmental disorders. Both proteome and metabolome, in addition to genetic coding, can be influenced by environmental exposures so subtle variations between individuals can result in large perturbations of metabolite and protein expression; thus, the interplay between them could confound results. To overcome potential confounding effects, we used the health-Promoting Lifestyle Profile II (Walker et al., 1987), a valid, reliable, 52-item self-report questionnaire composed of six subscales comprehensive measure of lifestyle, nutrition, physical activity, health responsibility, spiritual growth, interpersonal relations, and stress management. This approach has been used in longitudinal studies of several diseases (Callaghan, 2005; Driver et al., 2006; Stuifbergen et al., 2006; Thanavaro et al., 2006). However, due to the limitation of the small sample size, and potential medication effect, further studies with larger sample sizes and more robust medication history are required to test the initial findings and elucidate and confirm the role of the potential identified biomarkers. In the future studies, we will use a questionnaire such as the Health-Promoting Lifestyle Profile-II to measure environmental exposures that may be confounders to proteome and metabolome results. So, the correlation of this molecular dysregulation with clinical phenotypes will help in developing a long-term stable strategy of detecting these biomarkers for developing high-risk neurocognitive deficits in 22qDS by using the power of high-throughput omics and molecular biology, as a novel model, to better understand the complex biology of 22qDS.

### Supplementary Information

Below is the link to the electronic supplementary material.

Supplementary file1 (XLSX 110 KB)

## Data Availability

The datasets generated during and/or analyzed during the current study are available from the corresponding author on reasonable request.
